# Thin-Film Composite Membrane with Porous Interlayer Composed of Dendritic Mesoporous Silica Nanoparticles for Enhanced Nanofiltration

**DOI:** 10.3390/polym15193912

**Published:** 2023-09-28

**Authors:** Chi Jiang, Mengmeng Zhang, Yingfei Hou

**Affiliations:** 1 Institute of Carbon Neutrality, College of Chemical and Biological Engineering, Shandong University of Science and Technology, Qingdao 266590, China; 2 State Key Laboratory of Heavy Oil Processing, College of Chemistry and Chemical Engineering, China University of Petroleum (East China), Qingdao 266580, Chinahouyf@upc.edu.cn (Y.H.)

**Keywords:** nanofiltration, thin-film composite membrane, interlayer, dendritic nanoparticle

## Abstract

Positively charged nanofiltration (NF) membranes show great potential in the fields of water treatment and resource recovery. However, this kind of NF membrane usually suffers from relatively low water permeance. Herein, a positively charged NF membrane with a porous interlayer is developed, where the interlayer is formed by assembling dendritic mesoporous silica nanoparticles (DMSNs) after the formation of a polyamide layer. This post-assembly strategy avoids the adverse effect of the interlayer on the formation of positively charged NF membranes. The porous DMSN interlayer provides abundant connected channels for water transport, thus endowing the NF membrane with enhanced water permeance. A series of DMSNs with different sizes was synthesized, and their influence on membrane formation and membrane performance was systematically investigated. The optimized membrane exhibits a CaCl_2_ rejection rate of 95.2% and a water flux of 133.6 L·h^−1^·m^−2^, which is 1.6 times that of the control group without an interlayer. This work represents an approach to the fabrication of a positively charged NF membrane with porous interlayers for high-efficiency cation rejection.

## 1. Introduction

With the continuous growth of the population and the increasingly serious water pollution problem, the shortage of freshwater resources has become an important factor threatening the sustained development of humanity [1,2]. Therefore, it is urgent to develop sustainable water treatment technologies. Nanofiltratoin (NF), as a typical pressure-driven membrane separation technology, has the advantages of easy operation and high efficiency. It has become an effective means to cope with the current water resource problems [3,4]. As the core of NF technology, NF membranes directly determine separation efficiency. Currently, the dominant NF membrane is the polyamide thin-film composite (TFC) membrane, which is prepared via interfacial polymerization (IP) between acyl chloride and amine on top of a porous support.

The NF separation process is mainly determined by the synergistic effect of steric hindrance and electrostatic repulsion. According to fixed surface charges on NF membranes, they are mainly divided into positively charged NF membranes and negatively charged NF membranes. Among them, positively charged NF membranes have a higher removal rate for multivalent cations such as Ca^2+^ and Mg^2+^, showing great potential in the fields of water softening and resource recovery (e.g., lithium extraction from salt lakes). In particular, with the explosive growth in demand for lithium resources, the research on positively charged NF membranes has received much attention [5,6]. Positively charged NF membranes can be fabricated by promoting the trans-interface diffusion of amine monomers, applying polyethyleneimine (PEI) to replace piperazine (PIP), and modifying the membrane surface using charged materials [7,8,9,10,11,12]. However, positively charged NF membranes usually suffer from relatively low water permeance, which leads to relatively high energy consumption. Thus, a highly permeable positively charged NF membrane is needed [5,13].

In recent years, researchers have introduced polymer, nanoparticle, and nanocomposite interlayers between porous supports and polyamide layers to improve the separation performance of TFC membranes [14,15,16,17]. The mechanisms of the interlayer strategy mainly include (1) regulating the formation of polyamide by influencing the diffusion reaction of amine monomers and (2) providing transport channels for water molecules collected by the polyamide layer (known as the “gutter effect”). Building an interlayer before IP is an effective strategy to improve NF membrane performance, particularly its water performance. However, these interlayer works focus on negatively charged NF membranes. The restricted diffusion of amine monomers caused by interlayers is unfavorable for the formation of positively charged polyamide layers. In addition, the conventional interlayer built by polymers or nanomaterials is still relatively dense. A porous interlayer is expected to provide a multi-channel, low-resistance gutter, which is in favor of achieving further improvement in water permeance.

Dendritic mesoporous silica nanoparticles (DMSNs) are a kind of novel porous material with a unique central radial pore structure. They have a three-dimensional mesoporous structure, adjustable pore size, large specific surface area, good hydrothermal stability, and good biocompatibility. Therefore, they have been widely used in various fields such as medicine [18,19], biomedicine [20,21], and catalysis [22]. However, there are few reports on their application in membranes for water treatment.

**Figure 1:**
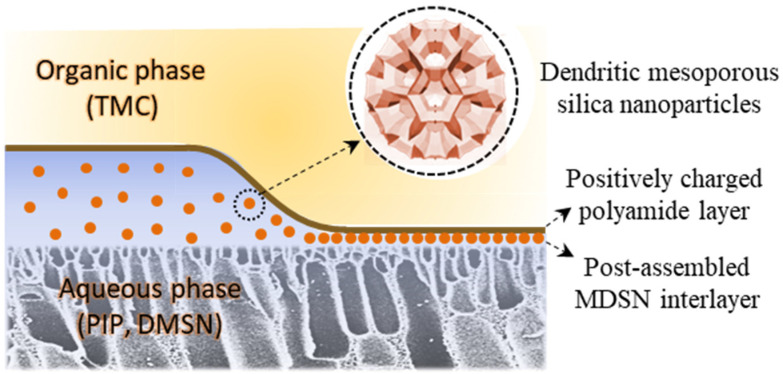
Illustration of TFC NF membrane with post-assembled MDSN interlayer.

In this work, a positively charged NF membrane with a porous DMSN interlayer is developed based on our proposed in situ free interfacial polymerization [23,24] (Figure 1). Different from conventional fabrication methods of interlayers, the DMSN interlayer is assembled after the formation of polyamide layer, avoiding the adverse effect on the formation of positively charged NF membranes. The porous DMSN interlayer provides abundant connected channels for water transport, thus endowing the NF membrane with enhanced water permeance. A series of DMSNs with different sizes were synthesized, and their structural properties were characterized. The influence of DMSN size and interlayer fabrication parameters on NF membrane performance was systematically investigated. This work represents an approach to the fabrication of positively charged NF membrane with porous interlayers for high-efficiency cation rejection.

## 2. Materials and Methods

### 2.1. Chemicals and Materials

The commercial polysulfone (PSF) ultrafiltration membrane, used as a support layer for TFC membrane preparation, was purchased from Beijing OriginWater Membrane Technology Co., Ltd. (Beijing, China) Tannic acid (TA, 99%), ferric chloride hexahydrate (FeCl_3_·6H_2_O, 99%), sodium dodecyl sulfate (SDS, 99%), tetraethyl orthosilicate (TEOS, 99%), triethylamine (TEA, analytical grade), cetyltrimethylammonium tosylate (CTA·Tos), 1-butyl-3-methylimidazolium trifluoromethanesulfonate ([BMIM]OTf), 3-aminopropyltriethoxysilane (APTES), and poly (ethylene glycol)-block-poly (propylene glycol)-block-poly (ethylene glycol) (F127), were purchased from Aladdin (Shanghai, China). Trimesoyl chloride (TMC, 98%) and piperazine (PIP, 98%) were purchased from TCI (Tokyo, Japan). Anhydrous sodium sulfate (Na_2_SO_4_), calcium chloride (CaCl_2_), sodium chloride (NaCl), xylene, and hexane were purchased from the Sinopharm Chemical Reagent Co., Ltd. (Shanghai, China). Deionized water was prepared using a two-stage reverse osmosis system.

### 2.2. Synthesis of DMSNs with Different Sizes

DMSNs with different sizes were synthesized according to the methods reported in the previous literature [25]. The reaction occurred at atmospheric pressure with a templating sol–gel technique using CTA·Tos, [BMIM] OTF and F127 as surfactants, TEA as a mineralizing agent, water as a solvent, and TEOS as a silica source. The tuning of the size of DMSNs was achieved by changing the type and concentration of the surfactants.

To synthesize DMSNs with diameter of 250 nm, CTA·Tos (0.96 g), TEA (0.105 g), [BMIM] OTF (300 mg), and water (50 mL) were stirred at 80 °C for 1 h, and then, TEOS (7.8 mL) was rapidly added to the mixture. The mixture was further stirred at 1000 rpm at 80 °C for 2 h. The resulting product was centrifuged and washed with ethanol and water, followed by drying in a vacuum oven at 80 °C for 8 h. The obtained DMSNs (1.0 g) were added to an ethanol/hydrochloric acid solution (ethanol (100 mL) and concentrated HCl (15 mL)) and ultrasonicated for 2 h, followed by stirring at 70 °C for 24 h. To effectively remove the surfactants from the surface of the DMSNs, the washing process was repeated three times. Finally, the precipitate was centrifuged and washed with ethanol, and dried at 60 °C in a vacuum oven for 24 h.

To synthesize DMSNs with diameter of 100 nm, CTA·Tos (0.96 g), TEA (0.1735 g), and water (50 mL) were stirred at 80 °C for 1 h, and then, TEOS (7.8 mL) was rapidly added to the mixture. The remaining steps were the same as those for the subsequent preparation of 250 nm DMSNs.

To synthesize DMSNs with diameter of 50 nm, CTA·Tos (0.96 g), TEA (0.1735 g), F127 (2.117 g), and water (50 mL) were stirred at 80 °C for 1 h, and then, TEOS (7.8 mL) was rapidly added to the solution. The remaining steps were the same as the post-preparation process for 250 nm DMSNs.

### 2.3. Fabrication of NF Membrane with DMSN Interlayer

Interfacial polymerization of TMC and PIP was used to prepare the TFC NF membrane. The basic method of membrane preparation was in situ free interfacial polymerization, proposed in our previous works [23,24]. The characteristic of free interfacial polymerization, different from the conventional method, is that there is a continuous aqueous-phase layer existing between the organic phase and the support. During interfacial polymerization, amide monomers (i.e., PIP) diffuse from the aqueous phase into the organic phase, and then, react with acyl chloride (i.e., TMC) to form a crosslinked polyamide film. In order to form a positively charged NF membrane, xylene and a surfactant (i.e., SDS) were added to promote the trans-interface diffusion of the PIP monomer. To introduce the porous interlayer, a certain amount of DMSN was added into the aqueous phase, which was pre-sonicated to prevent the DMSNs from aggregating.

**Figure 2:**
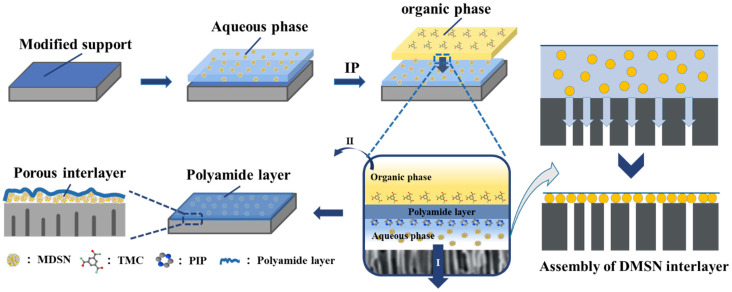
Preparation process of positively charged NF membrane with post-assembled DMSN interlayer.

The fabrication process is presented in Figure 2. The commercial PSF UF membrane was hydrophilically modified in a freshly prepared TA-Fe mixed solution [23]. Then, the aqueous phase solution containing DMSNs (0.01~0.08%), PIP (0.5%), and SDS (0.05%) was poured onto the modified PSF membrane and soaked for 2 min, followed by pouring the organic phase solution (0.12% TMC in hexane/xylene) onto the membrane surface to initiate the interfacial polymerization reaction. Ten seconds later, the unreacted aqueous phase solution was removed via vacuum suction (step I shown in Figure 2). During this step, DMSNs deposited on top of the PSF membrane to form an interlayer with the loss of water, and the polyamide layer was then deposited on top of the DMSN interlayer to form a TFC structure. The remaining organic phase solution was poured off (step II shown in Figure 2) and the membrane surface was rinsed with n-hexane for 15 s. The resulting TFC membrane was thermally treated in a 60 °C oven for 5 min, and then, stored in a refrigerator at 0–5 °C.

The experiments were designed to optimize the performance of the TFC membrane and to explore the effect of the DMSN water-phase doping concentration and its size on the performance of the resulting membranes. The obtained positively charged NF membrane with a DMSN interlayer was named Dn-TFC-x (D represents DMSNs, n represents the size of the DMSNs, and x represents the concentration of DMSNs in aqueous phase, %). The control group, i.e., the TFC NF membrane without an interlayer, was named TFC-0.

### 2.4. Characterization of DMSNs

Scanning electron microscopy (SEM, JEOL 7900F, Tokyo, Japan) and transmission electron microscopy (TEM, JEM 1200EX, JEOL, Tokyo, Japan) measurements were used to observe the morphology of the synthesized DMSNs powder. To prepare the SEM sample, a small amount of the powder was evenly distributed on a sample stage coated with conductive adhesive, and then, gold-sputtered before observation. To prepare the TEM sample, a small amount of DMSN powder was dispersed in water through ultrasound, and a suitable amount of the obtained colloid solution was dropped onto a specialized copper mesh; the resulting sample was then air-dried and measured.

### 2.5. Characterization of DMSN-Interlayered NF Membranes

The chemical composition of the membranes was characterized via attenuated total reflectance Fourier transform infrared spectroscopy (ATR-FTIR, NICOLETTM iS10 spectrometer, Thermo Fisher Scientific, Waltham, MA, USA) and X-ray photoelectron spectroscopy (XPS, Quanta200 spectrometer, FEI, Hillsboro, OR, USA). The XPS results were then analyzed using CasaXPS software (2316PR1.6). The characterization of the morphology of the membrane material included the measurement and analysis of surface roughness and surface morphology using atomic force microscopy (AFM, SPM-9700, Shimadzu, Kyoto, Japan) and SEM, respectively. The cross-sectional structure of the membrane was characterized using SEM and TEM. We used the following protocols. AFM measurement: Clean the membrane piece and cut it to an appropriate size, and then, stick it to the AFM sample stage to scan the sample in tapping mode. Linearly fit and denoise the acquired images, and calculate the surface roughness of the sample, including the average roughness (R_a_) and root-mean-square roughness (R_q_). The tip radius is less than 10 nm, the cantilever length is 125 μm, and the force constant is 42 N/m. SEM cross-sectional testing: First, remove the bottom nonwoven fabric of the TFC membrane with tape, and then, put the remaining UF layer and separation layer in liquid nitrogen for brittle fracture. Take a smooth and flat cross-sectional part as the SEM test sample, and spray it with gold before testing. SEM surface testing: After the clean membrane piece is attached flat to the sample stage, spray it with gold, and then, test the surface morphology of the membrane.

### 2.6. NF Membrane Performance

The separation performance of the NF membrane (water permeance and salt rejection) was evaluated through a laboratory-manufactured cross-flow filtration device, which includes 6 test units with an effective membrane area of 17.7 cm^2^. The operating pressure was set at 1.0 MPa, and the testing temperature was maintained at 25 °C. Prior to measurement, all NF membranes were pressurized at a pressure of 1.0 MPa for 1 h to achieve a stable state. Then, the TFC NF membrane was tested for its permeation flux (*F*) and rejection rate (*R*) in response to different salts.

(1) $$F=\frac{V}{S\cdot T}\times 100\%$$

In the above equation, *F* is the water flux, L·m^−2^·h^−1^; *V* is the volume of collected water in a certain time, L; *T* is the time for collecting the passed liquid, hours; and *S* is the effective filtration area of the membrane, m^2^.

The rejection performance of the NF membrane was measured using a series of salt solutions (CaCl_2_, Na_2_SO_4_, NaCl, 2000 ppm), and the formula for calculating the retention rate is as follows:

(2) $$R=(1-\frac{C_{p}}{C_{f}})\times 100\%$$

where *R* is the rejection rate, %, and *C_p_* and *C_f_* are the salt concentration of permeated liquid and feed.

## 3. Results and Discussion

### 3.1. Characterization of the Synthesized DMSNs

**Figure 3:**
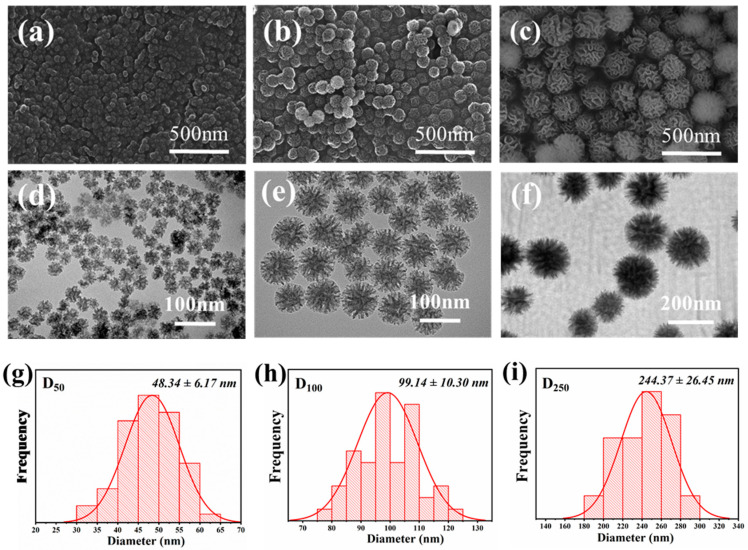
SEM and TEM images and size distribution of DMSNs with different particle sizes. (**a**–**c**) SEM images; (**d**–**f**) TEM images; (**g**–**i**) size distribution; (**a**,**d**,**g**) 50 nm DMSNs; (**b**,**e**,**h**) 100 nm DMSNs; (**c**,**f**,**i**) 250 nm DMSNs.

Three kinds of DMSNs with different sizes were synthesized. The micro-morphology and structural properties of the DMSNs were characterized through SEM measurement, and the results are presented in Figure 3. The synthesized DMSNs have a uniformly distributed size, and exhibit branched and wrinkled structures. The wrinkled sheets of silica lead to abundant pores within the DMSNs. The TEM images in Figure 3 show that all DMSNs possess three-dimensional central radiation pore channels and multi-level pore structures, which is consistent with the SEM characterization result. The sizes of the DMSNs were measured based on the TEM images, and the size distribution of the DMSNs was further determined from the TEM images using ImageJ software (1.8.0.345). The results (Figure 3g–i and Figure S2) indicate that the mean diameters of the three kinds of DMSNs are about 48 nm, 99 nm, and 244 nm, respectively, which are close to the expected values (i.e., 50, 100 and, 250). In addition, the results show that the wrinkled structures become looser and the opening degrees of the pores increase with the increase in the size of the DMSNs.

### 3.2. Physicochemical Properties of the Fabricated NF Membranes

**Figure 4:**
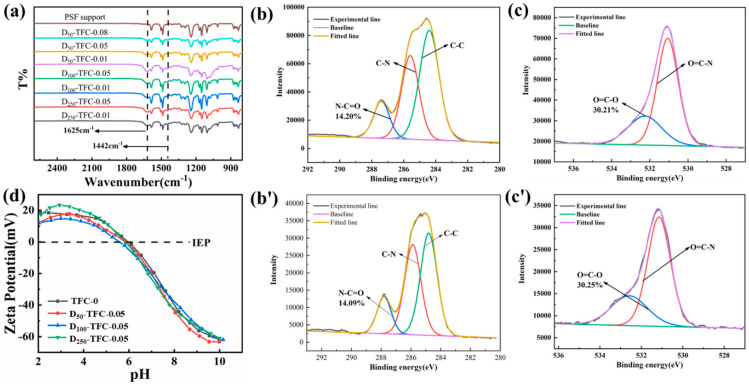
(**a**) ATR-FTIR spectra of the PSF support and the prepared TFC membranes; (**b**) C1s XPS spectrum and (**b′**) O1s XPS spectrum of TFC-0; (**c**) C1s XPS spectrum and (**c′**) O1s XPS spectrum of D_100_-TFC-0.05; (**d**) zeta potential of the prepared TFC membranes.

The TFC membranes were fabricated via IP. The chemical compositions of the support and the TFC membranes prepared under different conditions were characterized via ATR-FTIR. As shown in Figure 4a, compared with the support, the prepared TFC membranes all exhibit two new absorption peaks at 1625 cm^−1^ and 1442 cm^−1^; these peaks correspond to the stretching vibrations of carbonyl groups in the semi-aromatic amide bond, demonstrating the successful formation of a polyamide separation layer. The chemical composition of the TFC membranes was further characterized via XPS, and the high-resolution C1s and O1s spectra are presented in Figure 4b,c. The peaks of N-C=O, C-N, and C-C are observed in the C1s spectra, and the peaks of O-C=O and N-C=O are observed in the O1s spectra. The proportion of N-C=O in the C1s spectra is about 14%, and the proportion of O-C=O in O1s spectra is about 30%. The cross-linking degrees of the NF membranes fabricated with different DMSNs were calculated based on the XPS results, which shows that all the NF membranes have relatively high crosslinking degrees (Table S1 in Supplementary Material).

Comparing the interlayered TFC membranes with the control group (TFC-0), the type and proportion of functional groups in the polyamide separation layer are nearly unchanged. This indicates that the introduction of the DMSN interlayer did not affect the chemical composition of the polyamide layer. This point is also supported by the result of the zeta potential measurement. As presented in Figure 4d, the surface charge properties of the NF membranes with or without the DMSN interlayer are exactly the same (the membranes show a positive charge under a pH of higher than 6). The unchanged physicochemical properties of polyamide after introducing the interlayer are attributed to the following two reasons: (1) the formation of the DMSN interlayer occurred after the formation of the polyamide separation layer and (2) the membrane-forming reaction occurred at the interface between the two phases, while the DMSNs were uniformly dispersed in the bulk phase of the aqueous solution, and thus, did not interfere with the reaction.

### 3.3. Structural Properties of the Fabricated NF Membranes

**Figure 5:**
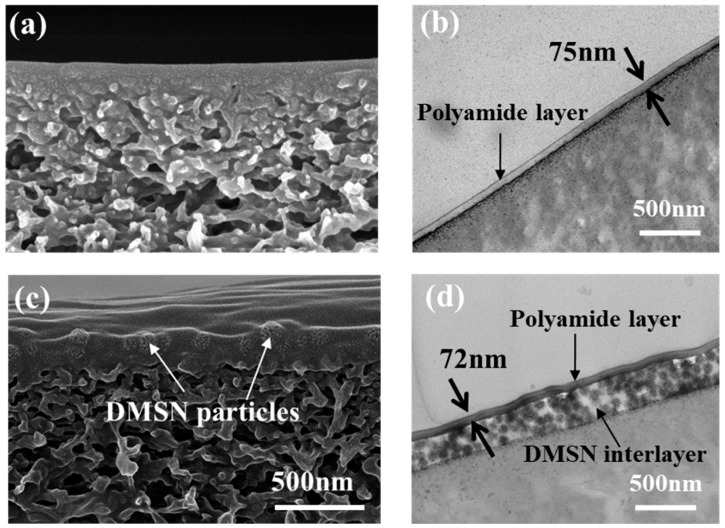
(**a**) Cross-sectional SEM image and (**b**) cross-sectional TEM image of TFC-0; (**c**) cross-sectional SEM image and (**d**) cross-sectional TEM image of D_100_-TFC-0.05.

To investigate the effect of the DMSN interlayer on the structural properties of the TFC membranes, the cross-sectional morphology of the TFC NF membranes with or without the DMSN interlayer was characterized through SEM and TEM measurements, and the results are presented in Figure 5. The presence of the interlayer is clearly observed in both the SEM and TEM images. After the introduction of the interlayer, a “sandwich” structure containing the support, the DMSN interlayer, and the polyamide separation layer is formed. Importantly, the formation of the DMSN interlayer results in abundant interspace between the separation layer and the support, which is expected to serve as the transport channel for water molecules and reduces the mass transfer resistance of water molecules permeating cross the TFC membrane. In addition, according to TEM measurement, the introduction of the DMSN interlayer does not affect the thickness and continuity of the polyamide layer (the thickness of the interlayer is about 75 nm).

**Figure 6:**
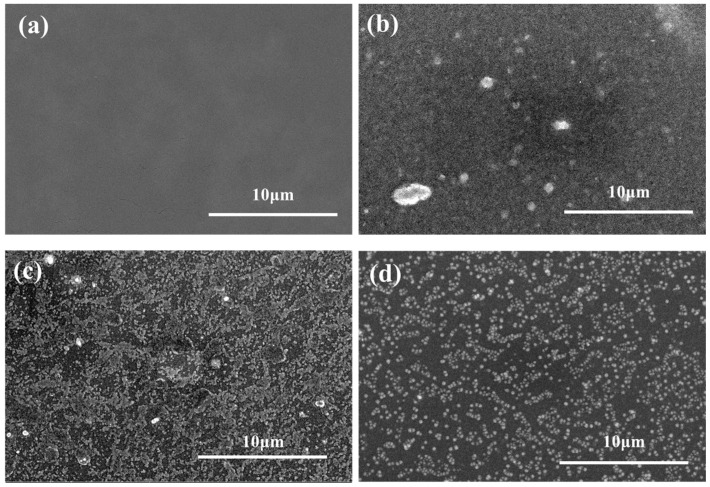
Surface SEM images of (**a**) TFC-0, (**b**) D_50_-TFC-0.03, (**c**) D_100_-TFC-0.03, and (**d**) D_250_-TFC-0.03.

**Figure 7:**
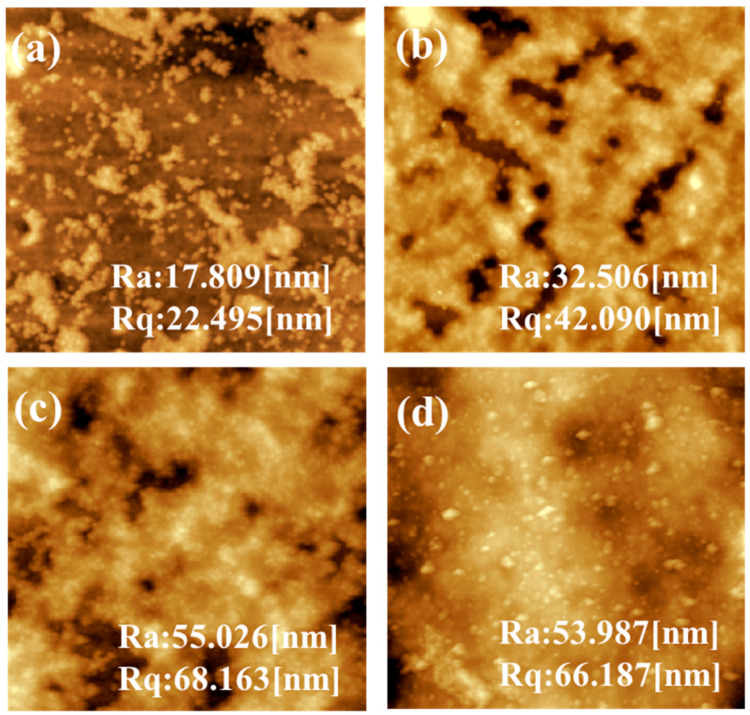
Surface AFM images of (**a**) D_100_-TFC-0.01, (**b**) D_100_-TFC-0.03, (**c**) D_100_-TFC-0.05, and (**d**) D_100_-TFC-0.08.

The surface SEM images of the TFC NF membranes with or without the DMSN interlayer are shown in Figure 6. The surface of the traditional TFC NF membrane (TFC-0) is relatively smooth, while the NF membranes with DMSNs of various sizes show rough surface morphologies, indicating that the introduction of DMSNs has a certain impact on the surface morphology of the polyamide layer, although they are located under the polyamide layer. The quantitative information about the influence of DMSN doping concentration on membrane surface roughness was further explored via AFM measurement. Figure 7 shows the surface AFM images of the TFC membrane prepared under different doping concentrations of 100 nm DMSNs. With the increase in doping concentration from 0.01% to 0.08%, the surface roughness (r_a_) of the TFC membranes increases from 17 nm to 53 nm. The increased roughness may originate from the aggregation of DMSN during the assembly process.

### 3.4. NF Membrane Performance

**Figure 8:**
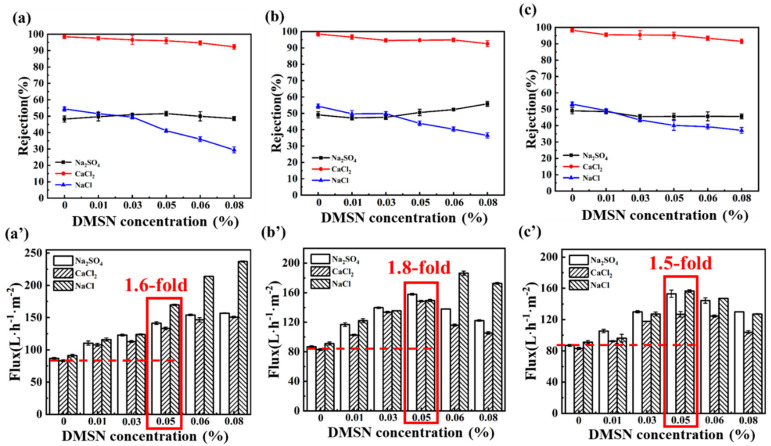
(**a**–**c**) Salt rejection performance and (**a′**–**c′**) water flux of TFC membranes fabricated with different DMSN addition amounts: (**a**,**a′**) D_50_-TFC, (**b**,**b′**) D_100_-TFC, (**c**,**c′**) D_250_-TFC.

A series of NF membranes were fabricated by varying the DMSNs’ sizes and their addition amounts in the formation of the interlayer. The separation performance of the fabricated NF membranes was evaluated via crossflow filtration tests, and the results are presented in Figure 8. Different influence laws of DMSNs’ concentration on the water permeance of the resulting NF membrane are observed for the three different systems. For the 50 nm DMSN system (Figure 8a′), the water flux of the TFC NF membrane increases from 83.1 L^−1^ m^−2^ h^−1^ to 150.7 L^−1^ m^−2^ h^−1^ with an increase in the DMSN addition concentration from 0 to 0.08%. However, for the 100 nm and 250 nm DMSN systems shown in Figure 8b′,c′, the water fluxes of the TFC membranes first increase, and then, decrease with increasing DMSN concentration, with the peak appearing at a 0.05% concentration.

**Figure 9:**
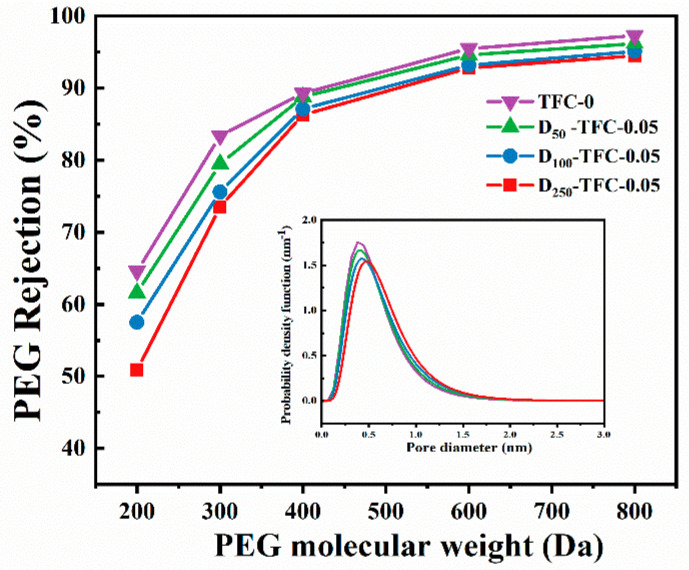
MWCOs and pore size distribution curves of the fabricated membranes.

As regards the rejection performance, the three systems show similar phenomena. The rejection rates of the resulting NF membranes follow the order of CaCl_2_ > Na_2_SO_4_ ≈ NaCl, agreeing well with the typical rejection property of the positively charged NF membrane. With the increase in the doping concentration of the DMSNs, the rejection rates of the NF membranes toward CaCl_2_ and NaCl_2_ slightly decrease, while the rejection rate toward Na_2_SO_4_ slightly increases. For example, the CaCl_2_ rejection rate of the D_100_-TFC membranes decreases from 98.4% to 92.6% when the DMSN concentration increases from 0 to 0.08%, while the Na_2_SO_4_ rejection rate slightly increases from 49.8% to 55.8%. This may be attributed to the following reasons: (1) although the DMSNs are located under the polyamide layer, the negatively charged property of the silicon hydroxyl group of DMSNs also influences the rejection performance for charged ions due to the Donnan effect; (2) the stacked DMSNs reduce the interaction between the polyamide layer and the support, which may lead to microscopic defects (the effect of steric hindrance is weakened). In addition, the molecular weight cut-offs (MWCOs) of the membranes were evaluated by filtrating the neutral solutes (i.e., PEG) with different molecular weights, and the pore size distributions of the fabricated membranes were further estimated. The result (Figure 9) indicates that the introduction of DMSNs leads to a slightly increased MWCO value. Correspondingly, the calculated pore size distribution of the fabricated membranes slightly increases, which is in line with the slightly reduced rejection toward Ca^2+^.

After comprehensive consideration of the salt rejection and water flux of the TFC membranes for the three salts solutions, the same optimal concentration is obtained for different sizes of DMSNs: 0.05%. Although the optimal doping concentrations are same for different systems, the improvement effects of the DMSN interlayer on water permeance are different. At this optimal doping concentration, the interlayered TFC NF membrane with DMSNs of 50 nm exhibits a CaCl_2_ rejection rate of 95.2% and a water flux of 133.6 L·h^−1^·m^−2^, which is 1.6 times that of the control group without the interlayer. The interlayered TFC NF membrane with DMSNs of 100 nm exhibits a CaCl_2_ rejection rate of 94.7% and a flux of 148.5 L·h^−1^·m^−2^, which is 1.8 times that of the control group. The interlayered TFC NF membrane with DMSNs of 250 nm has a CaCl_2_ rejection rate of 95.0% and a water flux of 128.7 L·h^−1^·m^−2^.

### 3.5. Mechanism and Performance Comparison

**Figure 10:**
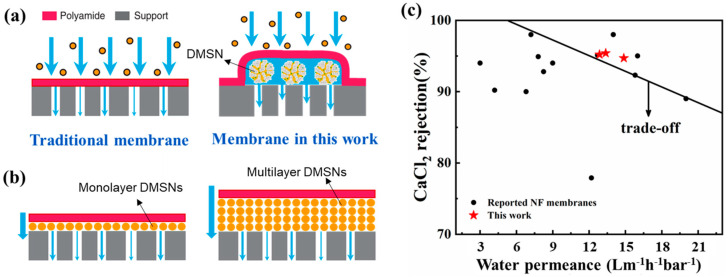
(**a**) Mechanism of the enhanced water flux for DMSN-interlayered TFC membrane; (**b**) deposition of excess DMSNs leads to increased diffusion distance for water permeating cross TFC membrane; (**c**) comparison of the separation performance between our optimized NF membrane and the positively charged NF membranes reported in the literature.

The introduction of DMSNs leads to the formation of a porous interlayer between the polyamide layer and the support layer, which provides low-resistance transport channels for water collected by the polyamide layer. Thus, the interlayer increases the water transport rate and results in higher water flux (Figure 10). The effect of the interlayer has been discussed in the previous literature [16,26]. For this work, the properties of the interlayer are influenced by the addition concentration and size of the DMSNs. The addition concentration determines the continuity and thickness of the interlayer. Too low an addition concentration of DSMNs leads to a discontinuous interlayer, while the deposition of excess DMSNs leads to a thicker interlayer composed of multi-stacked DMSNs, which adversely leads to an increased diffusion distance for water permeating across the TFC membrane (Figure 10b). Thus, the water flux of the fabricated membranes does not monotonically increase with the addition amount of DMSNs. On the other hand, different DMSNs have different opening degree of their pores. The wrinkled structures become looser and the opening degrees of the pores increase with an increase in size of DMSNs, which influences the porosity of the formed interlayer. However, the increased diameter of the DMSNs also leads to an increased water transport distance. As a result, DMSNs with a moderate size (i.e., 100 nm) lead to the interlayered NF membrane with the best performance (in terms of water flux).

Furthermore, Figure 10c compares the TFC NF membranes prepared under the optimal conditions in this study with the positively charged NF membrane reported in the literature [27,28,29,30]. The comparison shows that our fabricated positively charged NF membrane with a DMSN interlayer has a significant advantage over the NF membranes reported in the literature in terms of both water permeance and CaCl_2_ rejection. The increased water flux can lead to reduced operation pressure and energy consumption of the membrane separation process, and the increased salt rejection gives it great application potential in the fields of water softening and drinking water production. However, the interlayer may reduce the adhesive strength between the polyamide layer and the support, which brings potential risks for practical application (a detailed discussion is provided in the Supplementary Material).

## 4. Conclusions

This work presents the preparation of positively charged NF membranes with a DMSN interlayer. The impact of the DMSN interlayer on the physicochemical and structural properties of the TFC membranes was analyzed through characterization techniques such as SEM, AFM, XPS, and TEM. The mechanism of DMSNs in enhancing permeation flux was explored, and the optimal DMSN doping concentration for different particle sizes was determined. The results showed that the introduction of the DMSN interlayer resulted in a “sandwich” structure for the TFC membrane. The polyamide separation layer maintained its integrity and continuity, and the thickness and surface charge property of the polyamide layer were almost unchanged. The introduction of a porous DMSN interlayer provided abundant low-resistance transmission channels for water molecules, thereby increasing the permeation flux of the TFC membrane. The optimized TFC membrane exhibited a 94.7% rejection rate for CaCl_2_, and a water permeance of 14.9 L·h^−1^·m^−2^·bar^−1^, which was 1.8 times that of the control group without an interlayer, which successfully broke the permeability–selectivity “trade-off” limit and had a significant performance advantage over positively charged NF membranes reported in the literature.

## Data Availability

Not applicable.

## Supplementary Materials

The following supporting information can be downloaded at: https://www.mdpi.com/article/10.3390/polym15193912/s1, Figure S1. Illustration of formation process of DMSNs; Figure S2. TEM images of different DMSNs; Table S1. Element content and calculated crosslinking degree of the fabricated membranes; Table S2. Performance of the optimized NF membranes. Reference [31] is cited in the supplementary materials.
