# Supplementary material for: Thin-Film Composite Membrane with Porous Interlayer Composed of Dendritic Mesoporous Silica Nanoparticles for Enhanced Nanofiltration
Source: Polymers (Basel). 2023 Sep 28;15(19):3912. doi: 10.3390/polym15193912 (PMC10574978; doi:10.3390/polym15193912)
Supplement: Supplementary file 1 [file polymers-15-03912-s001.zip › polymers-2330378-supplementary.pdf]

# Supplementary Material

## Thin-film composite membrane with porous interlayer composed of dendritic mesoporous silica nanoparticles for enhanced nanofiltration

Chi Jiang[*12], Mengmeng Zhang and Yingfei Hou[1]

1 Institute of Carbon Neutrality, College of Chemical and Biological Engineering, Shandong University of Science and Technology, Qingdao 266590, Shandong, China

2 State Key Laboratory of Heavy Oil Processing, College of Chemistry and Chemical Engineering, China University of Petroleum (East China), Qingdao 266580, China

1. Synthesis of dendritic mesoporous silica nanoparticles............................................2

2. Size distribution of the synthesized DMSNs ..........................................................3

3. Cross-linking degree of the fabricated NF membranes ............................................3

4. Pore size distribution of the fabricated NF membranes............................................4

5. Performance of the optimized NF membranes ........................................................5

6. Potential risks of the TFC membrane with DMSN interlayer ..................................5

# 1. Synthesis of dendritic mesoporous silica nanoparticles

In this work, dendritic mesoporous silica nanoparticles (DMSNs) were used to prepare the porous interlayer for TFC membrane. The synthesis of different DMSNs was based on the methods reported in the previous literature [*ACS Appl. Mater. Inter.* 2019,11: 10426-10433].

Mesoporous silica nanoparticles with controllable particle size attract much research interests in recent years. In particular, with a radiation-aligned mesopores in size below 200 nm were found to be applicable to biomedical and pharmaceutical fields. To date, great efforts have been put on the development of recipes for the synthesis of DMSNs. Most pathways for the production of DMSNs rely on the soft-templating strategy.

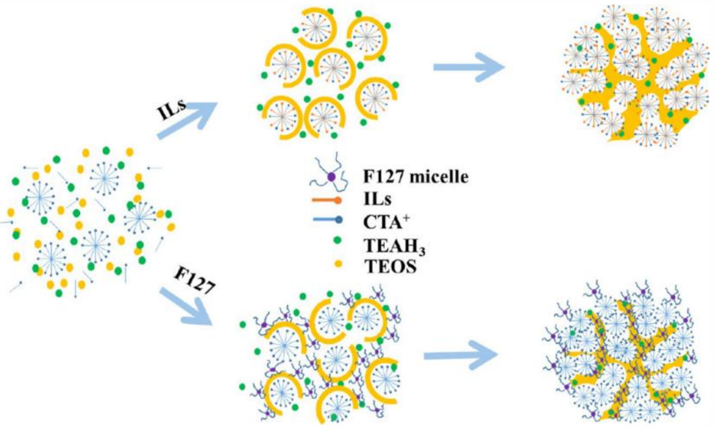


**Figure S1** Illustration of formation process of DMSNs

As presented in Figure S1, mesostructural surfactant-silica nanocomposites spontaneously assemble through interaction matching of the organic and inorganic components. In addition to thermodynamics of the surfactant–silica assembly, the morphologies and dimensions of the resulting materials are particularly dependent on the kinetics of sol–gel chemistry (such as the reaction temperature, water content, and pH value of the reaction solution). With a careful control of the self-assembly and silica condensation rate, it is possible to tailor the sizes, mesostructures and morphologies of the mesoporous silicas. The silica condensation reaction is shown as following:

$$-Si-O^{-} + HO-Si- \rightarrow -Si-O-Si- + OH^{-}$$

In this work, DMSNs were synthesized using cetyltrimethylammonium tosylate (CTA·Tos) ,1-butyl-3-methylimidazolium trifluoromethanesulfonate ([BMIM] OTF) and F127 as surfactants, triethanolamine (TEA) as a mineralizing agent, water as a solvent and tetraethyl orthosilicate (TEOS) as a silica source. The tuning of size of DMSNs was achieved by changing the type and concentration of surfactants.

## 2. Size distribution of the synthesized DMSNs

The size distribution of the DMSNs was further determined from the TEM images using Image J software.

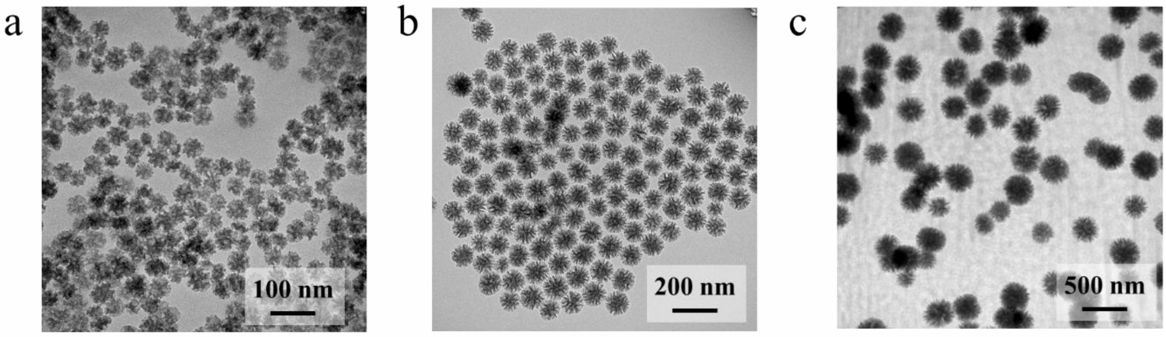


**Figure S2** TEM images of different DMSNs.

## 3. Cross-linking degree of the fabricated NF membranes

The cross-linking degree of the NF membranes fabricated with different DMSNs was calculated based on the XPS result, according to the following equations:

$$\text{Calculated crosslinking degree (\%)} = \frac{m}{m+n} \times 100\% \qquad \text{(S1)}$$

$$m+n=1 \qquad \text{(S2)}$$

$$\frac{\text{O}}{\text{N}} = \frac{3m+4n}{3m+2n} \qquad \text{(S3)}$$

The fully cross-linked structure infers that each acid chloride group (Cl–C=O) of TMC is bonded to an amino group of amine monomer forming an amide structure and hence every TMC is linked with 3×1/2 amine monomers. In the case of a fully linear structure one acid chloride group of TMC remains unreacted and is finally hydrolyzed to form a carboxylic acid. Thus there is a good relationship between O/N ratio and

crosslinking degree, which is described as equation R3. The O/N ratio can be obtained by XPS measurement.

**Table S1** Element content and calculated crosslinking degree of the fabricated membranes

| Membranes | C(%) | O(%) | N(%) | n[O]/n[N] | Cross-linking degree (%) |
|---|---|---|---|---|---|
| TFC-0 | 70.63 | 14.83 | 14.54 | 1.02 | 95.6 |
| $D_{50}$-TFC-0.05 | 70.33 | 15.18 | 14.49 | 1.05 | 92.7 |
| $D_{100}$-TFC-0.05 | 72.13 | 14.31 | 13.56 | 1.06 | 91.3 |
| $D_{250}$-TFC-0.05 | 72.00 | 14.41 | 13.59 | 1.06 | 91.3 |

## 4. Pore size distribution of the fabricated NF membranes

The MWCOs of the membranes were evaluated by filtrating neutral solutes (i.e. PEG) with different molecular weight. The pore size distributions of the fabricated membranes were further estimated by solute transport model based [*J. Membr. Sci.*, 1998, 142: 111-127]. Since it's a linear relationship between solute rejection ($R$) and solute diameter ($d_s$) on a log-normal probability paper, the mean solute size can be calculated as $d_s$ corresponding to $R$=50%. By ignoring the dependence of solute rejection on the steric and hydrodynamic interaction between solute and pore sizes, the mean pore size ($\mu_p$) can be considered to be the same as mean solute size. And the geometric standard deviation ($\sigma_p$) of the membrane can be determined from the radio of pore size at $R$=84.13% to that at $R$=50%. After obtaining $\mu_p$ and $\sigma_p$, the pore size distribution of the membrane can be plotted according to following probability density function.

$$\frac{\mathrm{d}f(d_p)}{d_p} = \frac{1}{d_p \ln\sigma_\mathrm{p}\sqrt{2\pi}} \exp\left[-\frac{(\ln d_\mathrm{p} - \ln\mu_\mathrm{p})^2}{2(\ln\sigma_\mathrm{p})^2}\right]$$

s

## 5. Performance of the optimized NF membranes

**Table S2** Performance of the optimized NF membranes

| Membranes | Water permeance ($Lm^{-2}h^{-1}bar^{-1}$) | $CaCl_2$ rejection (%) |
|---|---|---|
| $D_{50}$-TFC-0.05 | 13.4 | 95.2% |
| $D_{100}$-TFC-0.05 | 14.9 | 94.7% |
| $D_{250}$-TFC-0.05 | 12.9 | 95.0% |

## 6. Potential risks of the TFC membrane with DMSN interlayer

The increased water permeance is originated from the porous interlayer composed of DMSNs. However, the interlayer may reduce the adhesive strength between polyamide layer and support. For conventional TFC membrane, there is physical riveting between polyamide active layer and support due to the intrusion of polyamide into support pores. In this work, the interlayer avoided the intrusion, inescapably weakening the interaction force between the two layers. However, this is a common problem for the interlayered TFC membrane. Actually, although losing the physical riveting between polyamide layer and support pores, the composite structures are still enough stable during our filtration test, which may be attributed to the positive test pressure and intermolecular interaction between separation layer, interlayer and supporting layer.
